# Transient Ischemic Attack Due to Unruptured Basilar Artery Aneurysm

**DOI:** 10.7759/cureus.24102

**Published:** 2022-04-13

**Authors:** Vivek Bhat, Suresha Kodapala

**Affiliations:** 1 Internal Medicine, St. John's Medical College, Bangalore, IND; 2 Neurology, Vydehi Institute of Medical Sciences and Research Centre, Bangalore, IND

**Keywords:** tia, subarachnoid hemorrhage, antiplatelet therapy, endovascular aneurysm repair, aneurym, cerebrovascular disease, hemiplegia, stroke

## Abstract

Intracranial aneurysms are typically asymptomatic. They are usually incidentally detected or detected only after rupture. Ischemic stroke or transient ischemic attack (TIA) due to unruptured intracranial aneurysms (UIAs) is rare.

A 79-year-old male with well-controlled hypertension and hypothyroidism, presented with complaints of sudden-onset weakness of the right upper limb and lower limb, followed by altered sensorium and a fall. Two hours later, he had fully recovered. Neurologic examination was unremarkable. Computed tomography of the brain revealed a dilated and tortuous basilar artery, suggestive of an aneurysm compressing the left midbrain and pons, with no evidence of intracranial bleed. Further, magnetic resonance imaging with an angiogram revealed multiple lacunar infarcts in the posterior circulation, distal to the aneurysm. Finally, a cerebral angiogram confirmed a partially thrombosed, fusosaccular aneurysm, arising from the left vertebral and basilar arteries. In view of frailty and long vessel segment involvement, surgery was not advised. He was treated medically, with appropriate antiplatelets and prophylactic antiepileptics. On follow-up, he had no neurologic deficit and had suffered no later ischemic or hemorrhagic events.

UIAs may cause brainstem strokes via thrombosis of the parent vessel, emboli from the thrombus, or compression of the parent artery. In our case, compression, the least common mechanism, appears to have caused the TIA, with emboli potentially responsible for the silent lacunar infarcts. Fusiform aneurysms of the vertebrobasilar system have a poor natural history. In elderly patients presenting with ischemic events due to UIAs of the vertebrobasilar system, surgical intervention can be risky. So, medical treatment with antiplatelets is recommended.

UIAs should be considered in the differential diagnosis of patients with TIAs, and such patients should have a visualization of intracranial arteries.

## Introduction

Unruptured intracranial aneurysms (UIAs) are usually asymptomatic and noticed incidentally. If symptoms are present, they are usually secondary to mass effect, leading to headache, dizziness, or cranial nerve palsies [1]. Ischemic presentation - either ischemic stroke or transient ischemic attack (TIA) - is uncommon.

Literature on UIAs causing ischemic events is limited. We add to the available literature by reporting the case of an elderly male, who presented with transient neurologic deficits, that were later found to be due to a UIA of the vertebrobasilar system.

## Case presentation

A 79-year-old male, with well-controlled hypertension and hypothyroidism, presented to our center with a history of sudden-onset weakness of the right upper limb and lower limb. Following this, he briefly altered the sensorium and fell to the ground. He reported complete recovery within two hours of symptom onset. There was no history of loss of consciousness, seizures, facial deviation, altered vision, or smell. He had no prior history of headaches. He had no history of similar episodes in the past. He was not a known case of diabetes or dyslipidemia, nor did he have any history suggestive of ischemic heart disease. He was a non-smoker and did not consume alcohol.

On physical examination, his heart rate was 101 beats per minute and blood pressure was 120/89 mm Hg. He was conscious, cooperative, and oriented to time, place, and person. His extraocular movements were intact, there was no facial or tongue deviation, and his gag reflex was intact. Motor and sensory examinations were unremarkable. His history and examination, at this point, were suggestive of a TIA.

His basic hematologic and biochemical investigations were largely within normal limits, as summarized in Table 1.

**Table 1:** Basic hematologic and biochemical laboratory investigations at admission TLC - total leukocyte count; HDL - high-density lipoprotein; LDL - low-density lipoprotein; VLDL - very-low-density lipoprotein

Investigation	Patient value	Reference value
Hemoglobin	13.5 g/dL	13.0-17.0 g/dL
TLC	6.9 x 10^9^/L	4.0-11.0 x 10^9^/L
Platelets	30.3 x 10^9^/L	15-41 x 10^9^/L
Total cholesterol	130 mg/dL	<200 mg/dL
Triglycerides	145 mg/dL	<150 mg/dL
HDL	38 mg/dL	40-60 mg/dL
LDL	63 mg/dL	<130 mg/dL
VLDL	29 mg/dL	2-30 mg/dL
Glycated hemoglobin	6.4%	Normal - <5.6%, borderline - 5.6%-6.4%

**Figure 1:**
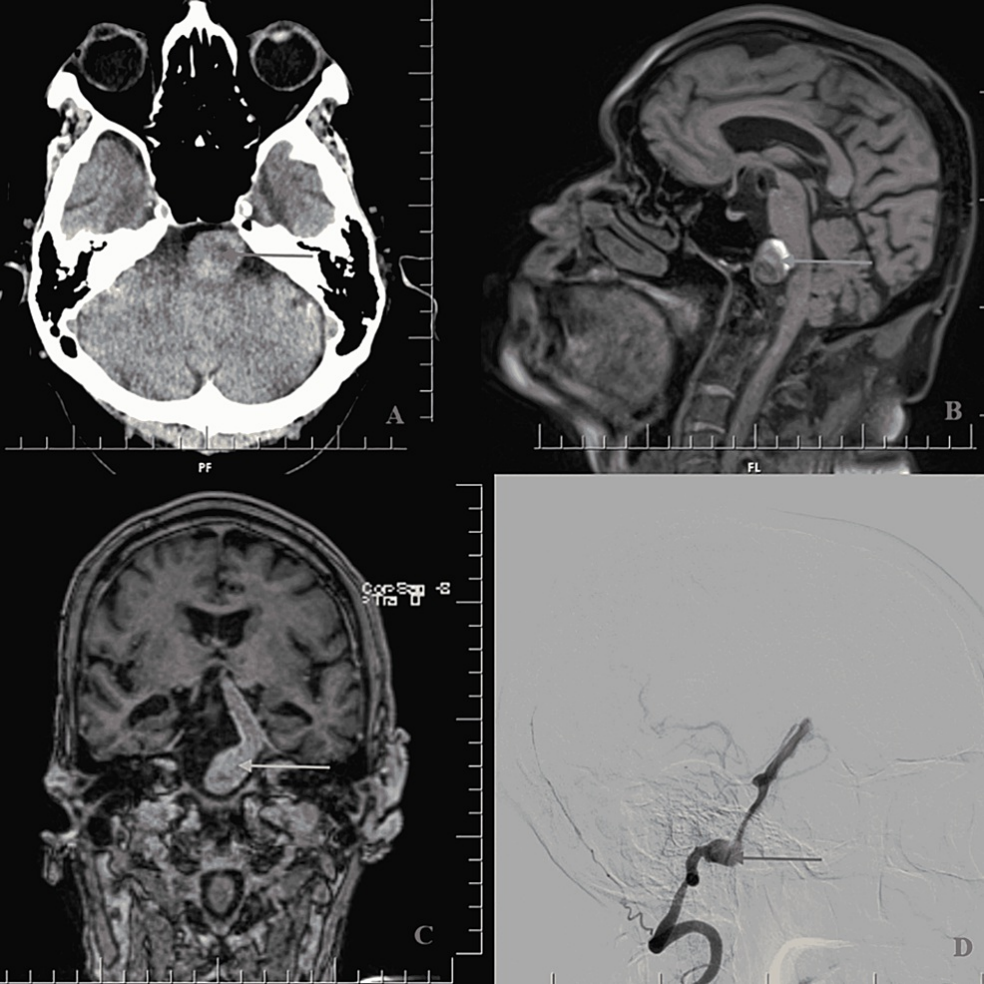
(A) CT brain axial section showing a dilated basilar artery, suggestive of an aneurysm, compressing the left pons (red arrow). (B) MRI brain sagittal section showing a partially thrombosed basilar artery aneurysm (blue arrow). (C) MRI brain coronal section showing a dilated basilar artery, and basilar artery aneurysm (yellow arrow). (D) Cerebral angiogram confirming a fusosaccular aneurysm arising from V3, V4 segments of left vertebral artery and basilar artery (purple arrow). CT - Computed tomography; MRI - Magnetic resonance imaging

We ordered relevant imaging of the brain and its vasculature. Computed tomography (CT) of the brain revealed a dilated and tortuous basilar artery, suggestive of an aneurysm, compressing the left midbrain and pons, with no evidence of intracranial bleed (Figure 1A). Magnetic resonance imaging (MRI) of the brain with angiogram revealed a partially thrombosed basilar artery aneurysm, with multiple, chronic lacunar infarcts in the posterior circulation distal to the aneurysm (Figures 1B, 1C). Finally, a formal cerebral angiogram confirmed a fusosaccular aneurysm, arising from the V3 and V4 segments of the left vertebral artery and basilar artery, with a maximum dimension of 1.7 cm (Figure 1D). With all these imaging findings, he was diagnosed with TIA due to an unruptured basilar artery aneurysm.

In view of his frailty and advanced age, the neurosurgeon and neuroradiologist did not advise surgical or endovascular intervention. He was discharged on appropriate dual antiplatelets, atorvastatin, and prophylactic antiepileptics - levetiracetam and clonazepam. On follow-up, he had no further complaints suggestive of an ischemic or hemorrhagic event.

## Discussion

UIAs are present in around 5% of the population [2]. They typically develop after the third decade of life and are more common in women, those with positive family history, and those with autosomal dominant polycystic kidney disease [2,3]. Most people with UIAs will spend their life without knowing of its presence.

In patients who suffer an ischemic stroke, UIAs are more commonly found than in the general population. This has been attributed to common risk factors for both stroke and UIAs - hypertension, diabetes, hyperlipidemia, and smoking [4,5]. However, rather than causing stroke, UIAs are typically incidental findings.

UIAs can, however, cause ischemic events. This is by three potential mechanisms - thrombosis and embolism from the aneurysm itself, thrombosis extending from the aneurysm to the parent vessel, or compression of the parent vessel [5]. Most reports of ischemic events in patients with UIAs have been attributed to thromboembolic phenomena, with compression rarely implicated [6]. In our patient, the TIA itself was probably due to compression of the basilar artery. Imaging revealed multiple silent, old lacunar infarcts, distal to the aneurysm, which we attribute to embolization.

UIAs involving the vertebrobasilar system are much less common than those involving the anterior circulation [7]. Broadly, based on their shape, they can be characterized as saccular and fusiform aneurysms. Fusiform aneurysms are less common than saccular aneurysms and are characterized by the presence of separate inflow and outflow ostia [8]. They have a greater risk of rupture, particularly with diameters greater than 1 cm, and greater morbidity [9]. Fusiform UIAs of the posterior circulation, such as in our patient, have some unique characteristics. Unlike UIAs overall, they have a male predisposition, are seen in typically older patients, and are more common in those with atherosclerotic risk factors [8]. Further, the pathophysiology of aneurysm formation here is different from that of the anterior circulation, due to the unique anatomy of the involved vasculature [8]. Finally, ischemic events are more common in vertebrobasilar UIAs compared to those in other locations. Patients with ischemic events as the presenting feature of vertebrobasilar aneurysms were more likely to recurrences compared to those without UIAs [8].

Ischemic events have been found to be a negative prognostic indicator in UIAs, with these patients having a greater risk of rupture and intracranial bleed [10]. The rate of enlargement of fusiform vertebrobasilar UIAs is also quite high, presenting an increased risk for rupture and brainstem stroke [9,11,12]. This highlights the need for appropriate management.

Vertebrobasilar aneurysms, particularly those with fusiform characteristics are known to be a treatment challenge [12]. Treatment modalities include microsurgery or endovascular interventions such as flow diversion or stenting. However, due to the advanced age and frailty of many of these patients, surgical or endovascular interventions may not be advisable in many of these patients [12-14]. In those patients presenting with ischemic events, medical management with antiplatelets has shown good results, with authors reporting reduced ischemic recurrences [8,12,15]. Our patient was advised against surgical intervention. He has done well, on solely medical management, for over a year since his initial presentation.

## Conclusions

UIAs are an underrecognized cause of ischemic stroke; they can rarely cause ischemic events due to parent vessel compression. Visualization of the intracranial vasculature with angiography should be considered in patients presenting with ischemic stroke or TIA to detect such aneurysms. In poor candidates for surgery, medical treatment with antiplatelets is effective.
